# The Carbon Dots from Seabuckthorn (*Hippophae rhamnoides* L.) Leaves: Recycle the Herbal Waste Products for a Nano-Formulation in Delivering Bioactive Compounds

**DOI:** 10.3390/jfb16120465

**Published:** 2025-12-17

**Authors:** Chen-Xi Xia, Xiong Gao, Queenie Wing-Sze Lai, Zheng-Qi Wang, Lish Sheng-Yin Lin, Janet Yuen-Man Ho, Jia-Yu Zhu, Roy Wai-Lun Tang, Tina Ting-Xia Dong, Karl Wah-Keung Tsim

**Affiliations:** 1 Shenzhen Key Laboratory of Edible and Medicinal Bioresources, HKUST Shenzhen Research Institute, Hi-Tech Park, Nanshan, Shenzhen 518000, China; cxiaad@connect.ust.hk (C.-X.X.); gaoxiong@ust.hk (X.G.); queenielai@ust.hk (Q.W.-S.L.); zwangie@connect.ust.hk (Z.-Q.W.); lishlin@ust.hk (L.S.-Y.L.); janetho@ust.hk (J.Y.-M.H.); jzhucg@connect.ust.hk (J.-Y.Z.); roytwl@ust.hk (R.W.-L.T.); botina@ust.hk (T.T.-X.D.); 2 Division of Life Science and Center for Chinese Medicine, The Hong Kong University of Science and Technology, Clear Water Bay, Hong Kong, China

**Keywords:** Seabuckthorn, carbon dot, flavonoid, drug encapsulation, bioavailability enhancement

## Abstract

Carbon dots have emerged as promising nanocarriers for drug delivery due to their unique physicochemical properties and biocompatibilities. Here, the potential of leaf-derived carbon dots (named as SBL_CD_), derived from Seabuckthorn (*Hippophae rhamnoides* L.), was illustrated as a novel nano-formulation for bioactive compound delivery. Seabuckthorn leaves, rich in flavonoids, are the waste product during the production of Seabuckthorn fruits. The wasted leaves were utilized to synthesize carbon dots via a hydrothermal method. The resulting SBL_CD_, characterized by TEM, FT-IR and Raman spectroscopy, exhibited a diameter of ~5 nm in both amorphous and quasi-crystalline forms. Applications of SBL_CD_ in cultures demonstrated robust properties of anti-inflammation and inducing neuronal cell differentiation. Furthermore, SBL_CD_ was able to encapsulate luteolin, a bioactive flavonoid. The enhanced delivery efficiency translated to superior biological activity, with SBL_CD_-luteolin requiring only 1.50 μg/mL in achieving the EC_50_ efficacy, as compared to 6.82 μg/mL for free luteolin in pNF200-Luc expression assays. This approach not only valorizes Seabuckthorn leaf by-products but also potentially improves the efficacy of encapsulated flavonoids. The development of SBL_CD_ as a multifunctional platform for flavonoid delivery represents a promising strategy in enhancing the efficacy of neuroactive compounds, combining anti-inflammatory effects (>70% cytokine suppression) with enhanced cellular uptake (4.5-fold increase).

## 1. Introduction

Carbon dots are fluorescent carbon-based nanoparticles with a size < 10 nm, which could be synthesized via top-down (e.g., laser ablation, arc discharge) or bottom-up (e.g., hydrothermal, microwave) methods. The synthesis of carbon dots combines facile, eco-friendly preparation with excellent biocompatibility, chemical inertness and low toxicity, making them versatile nanocarriers for drug delivery across cancer, ocular, infectious and central neuron system applications [1]. Biomass-derived carbon dots, prepared from plant leaves, fruit peels or agricultural residues via green hydrothermal or microwave strategies, offer sustainable, cost-effective routes to heteroatom-doped nanocarriers rich in surface functional groups, valorizing the waste products while minimizing environmental impact [2,3].

Flavonoids represent one of the most diverse groups of natural polyphenolic compounds, characterized by a basic C6-C3-C6 skeleton consisting of two aromatic rings (A and B) connected by a heterocyclic pyran ring (C) [4]. Flavonoids have diverse pharmacological activities through multiple molecular mechanisms, e.g., neuroprotective effects mediated through PI3K/Akt and MAPK signaling pathways, anti-inflammatory properties via inhibition of pro-inflammatory cytokines and antioxidant activities contributing to therapeutic potential in neurodegenerative diseases [5,6]. Despite the extensive pharmacological applications, flavonoids generally exhibit low bioavailability due to poor aqueous solubility and rapid metabolism [7]. Therefore, nanocarriers are expected to overcome these limitations by enhancing solubility, stability, permeability and controlled release [8,9]. In previous studies, quercetin-conjugated nitrogen-doped carbon dots have demonstrated enhanced cellular uptake and anti-cancer efficacy compared to free quercetin, underscoring carbon dots’ promise for flavonoid delivery [10]. However, no studies have explored whether agricultural waste-derived carbon dots can serve dual functions as both therapeutic agents and drug carriers, potentially eliminating the need for pharmacologically inert nanocarriers.

To address the concurrent challenges of agricultural waste management and low flavonoid bioavailability, this manuscript developed a novel approach that transforms discarded Seabuckthorn leaves into multifunctional carbon dots. Seabuckthorn (*Hippophae rhamnoides* L.) represents an exceptional natural resource rich in bioactive flavonoids, including isorhamnetin, quercetin and kaempferol, and other compounds that demonstrate significant neuroprotective potential [11]. The neuroprotective mechanisms of Seabuckthorn flavonoids involve the activation of critical signaling pathways, including PI3K/Akt and ERK cascades, that are essential for neuronal survival and differentiation, effectively mimicking neurotrophic functions and inducing neurite outgrowth in cultured neurons [12,13]. Indeed, Seabuckthorn flavonoids have been shown to restore depressive symptoms in mice [13]. Remarkably, Seabuckthorn leaves—typically discarded during fruit processing—contain 2~3-fold higher flavonoid levels than the commercially valuable fruit and yet remain underutilized. Thus, Seabuckthorn leaves represent an ideal candidate for high-value utilization in pharmaceutical applications while addressing the challenges of agricultural waste valorization [14].

Here, the leaves of Seabuckthorn were subjected to generate carbon dots. The characterization of Seabuckthorn leaf-derived carbon dots (SBL_CD_) was based on structure, optical properties, biocompatibility and neuroprotective activities in cultured neural cells. In addition, the drug-loading and delivery capabilities of SBL_CD_ by using luteolin as a model flavonoid were illustrated. The results support the applications of SBL_CD_ that can function as a dual-purpose nanomaterial, serving simultaneously as both a flavonoid-enriched neuroactive therapeutic agent and an efficient drug nanocarrier platform, thereby addressing the concurrent challenges of agricultural waste valorization and enhanced bioactive compound delivery.

## 2. Materials and Methods

### 2.1. Chemicals

Isorhamnetin, quercetin, kaempferol and luteolin of >98% purity were provided by Yuanye Biotechnology (Shanghai, China). Seabuckthorn leaves were collected from Qinghai, China. 3-(4,5-Dimethylthiazol-2-yl)-2,5-diphenyl tetrazolium bromide (MTT), Diphenylboric acid 2-aminoethyl ester (DPBA) and 4′,6-diamidino-2-phenylindole (DAPI) were purchased from Sigma-Aldrich (St Louis, MO, USA). Mito-Tracker Red CMXRos was bought from Beyotime Biotechnology (Shanghai, China). The pNF200-Luc plasmid was self-constructed. All cell culture-related reagents were obtained from Thermo Fisher Scientific (Waltham, MA, USA).

### 2.2. Hydrothermal Synthesis of Carbon Dots from Seabuckthorn Leaf (SBL_CD_)

The dried powders of Seabuckthorn leaf were passed through a 100-mesh screen to achieve fine particles. For hydrothermal synthesis, 5 g fine powder was dispersed in 100 mL deionized water in a 100 mL Teflon-lined autoclave, heated to 180 °C for 5 h in a blast air oven and then cooled naturally. The resultant was filtered through filter papers, centrifuged at 1500 rpm for 15 min and filtered through a 0.22 μm membrane. The obtained SBL_CD_ solution was dialyzed for 24 h against deionized water using a dialysis tube (500 Da MWCO; Sigma-Aldrich, St. Louis, MO, USA) and freeze-dried for storage.

### 2.3. Synthesis of Luteolin-Loaded SBL_CD_ (SBL_CD_-Luteolin)

Luteolin (2.86 μg) was dissolved in 1 mg/mL SBL_CD_ solution, ultrasonicated for 30 min and centrifuged through an ultrafiltration spin column (Amicon^®^ Ultra 0.5 mL centrifugal filters, 3 kDa MWCO; Merck, Darmstadt, Germany) at 14,000 rpm for 30 min to remove unbound luteolin. Loading optimization was performed by varying luteolin:SBL_CD_ ratios from 1:1 to 15:1 (*w*/*w*), and 2.86 μg luteolin per 1 mg/mL SBL_CD_ was selected as the optimal condition. The supernatant was centrifuged again at 14,000 rpm for 30 min to pelletize SBL_CD_-luteolin, which was then vacuum-dried overnight and stored at 4 °C in darkness.

### 2.4. Characterization of SBL_CD_ and SBL_CD_-Luteolin

Transmission electron microscopy (TEM) images were obtained using a Thermo Scientific Talos120c microscope (Thermo Fisher Scientific, Waltham, MA, USA) operated at 120 keV, with high-resolution images acquired by JEM-2010F FasTEM (JEOL Ltd., Tokyo, Japan). Particle size was calculated using ImageJ software (version 1.54g, NIH, Bethesda, MD, USA), and the size distribution was determined by ZetaView^®^ (Particle Metrix, Ammersee, Germany). FT-IR spectra were recorded using a VERTEX 70v FT-IR spectrometer (4000-500 cm^−1^) with Attenuated Total Reflectance (ATR) accessory (Bruker, Billerica, MA, USA). Raman spectroscopy was performed using an inVia™ Raman microscope (Renishaw, Gloucestershire, UK). UV-Vis absorption spectra were measured using a Shimadzu UV-2600i spectrophotometer (200–800 nm, 0.5 nm step size; Shimadzu, Tokyo, Japan). Photoluminescence spectra were recorded using HORIBA Fluorolog-QM™ spectrofluorometers (320–640 nm excitation range, 40 nm steps, 5 nm slit widths, 240 nm/min scan speed; HORIBA Scientific, Kyoto, Japan). XPS analysis was performed using a Kratos Axis Ultra DLD spectrometer equipped with a monochromatic Al Kα X-ray source (*hν* = 1486.6 eV) under ultra-high vacuum (<1.5 × 10^−7^ Pa) (Kratos Analytical Ltd., Manchester, UK). Survey spectra were acquired in low-resolution mode at a pass energy of 160 eV, while high-resolution C 1s, N 1s and O 1s spectra were recorded at a pass energy of 40 eV. Binding energies were calibrated using the adventitious C 1s *sp^2^* carbon peak at 285.0 eV as an internal reference.

### 2.5. HPLC-MS/MS Analysis

Chromatographic separation was performed using an Agilent ZORBAX Eclipse Plus C_18_ column (2.1 × 50 mm, 1.8 μm; Agilent Technologies, Waldbronn, Germany) with mobile phases of 0.1% formic acid in H_2_O (solvent A) and 0.1% formic acid in acetonitrile (solvent B). The gradient elution was 0–20 min, 2% B; 20–20.01 min, 100% B; and 20.01–25 min, 2% B. Injection volume was 5 μL, with a 0.3 mL/min flow rate. Multiple reaction monitoring (MRM) quantification used an Agilent Triple Quadrupole MS/MS (6410A; Agilent Technologies, Waldbronn, Germany) with ESI capillary voltage of 4000 V, drying gas temperature of 325 °C, gas flow of 10 L/min and nebulizer pressure of 35 psi. Transition ions for active compounds were optimized as listed in Table S1.

### 2.6. Cell Cultures and Viability

All cell cultures were maintained in a humidified incubator at 37 °C with 5% CO_2_. Rat pheochromocytoma PC12 cells (passage 15~25) were cultured in DMEM supplemented with 6% fetal bovine serum (FBS) and 6% horse serum. BV2 cells (passage 10~20) were cultured in DMEM supplemented with 10% heat-inactivated FBS. Cells were seeded in 96-well plates at a density of 1 × 10^4^ cells/well and treated with test compounds. MTT solution (0.5 mg/mL in basal DMEM) was added and incubated for 4 h. MTT solution was replaced with DMSO for cell lysis, and absorbance was measured at 570 nm using a Varioskan™ LUX multimode microplate reader (Thermo Fisher Scientific, Waltham, MA, USA). Cell viability was calculated by normalizing readings to the control group after blank subtraction.

### 2.7. Cytokine Measurement

The anti-inflammatory effects of SBL_CD_ were evaluated in mouse microglial BV2 cells. Briefly, cultured BV2 cells were pretreated with different doses of SBL_CD_ (5, 10, 20, 50, 100 μg/mL) or dexamethasone (10 μM, a positive control) for 2 h, followed by LPS challenge (20 ng/mL) for inducing neuronal inflammation. Total RNA was isolated from treated BV2 cells using RNAzol RT reagent (Molecular Research Center, Cincinnati, OH, USA) following the manufacturer’s protocol. In brief, cells were lysed by adding 1 mL RNAzol RT per 10 cm^2^ culture area, homogenized by gentle pipetting and incubated at room temperature for 5 min. After adding RNase-free water (0.4 mL per 1 mL reagent), the mixture was vortexed for 15 s, allowed to stand for 15 min and centrifuged at 12,000× *g* for 15 min at 4 °C. The RNA-containing supernatant was transferred to a fresh tube, and RNA was precipitated by adding an equal volume of 75% ethanol (0.4 mL per 1 mL supernatant), incubating for 10 min at room temperature and pelleting at 12,000× *g* for 8 min at 4 °C. After two washes with 75% ethanol, the RNA pellet was briefly air-dried and resuspended in RNase-free water. cDNA was synthesized by using the TaKaRa reverse transcription kit (TaKaRa Biotechnology, Dalian, China), and qRT-PCR was carried out on a Roche LightCycler 480 II instrument with SYBR Green chemistry. Each 10 µL reaction contained 5 µL 2× SYBR Green master mix, 1 µL cDNA, 0.3 µL each of 10 µM forward and reverse primers and 3.4 µL PCR-grade water. Amplification comprised 45 cycles of 95 °C for 30 s, 57 °C for 30 s and 72 °C for 30 s, and relative transcript levels were determined by the ΔΔ*Ct* method. Primer sequences are detailed in Table S2.

### 2.8. DNA Construct Transfection and Luciferase Assay

PC12 cells were seeded in a 24-well plate for pNF200-Luc transfection, with jetPRIME used for the transient plasmid transfection. The transfection procedure was conducted following the manufacturer’s instructions. Medium with transfection reagent was replaced after 4 h of transfection, followed by another 24 h of drug treatment at different doses in PC12 cells. Then, cell lysates were prepared for the luciferase assay as previously described [15]. After cultured medium was removed, cells were washed by PBS and lysed by using lysis buffer (100 mM PBS added with 1 mM DTT and 0.2% Triton X-100, pH 7.8). The lysate was collected, vortexed and centrifuged at 4 °C (12,000× *g*, 10 min). The supernatant was transferred to a white luciferase assay plate for enzymatic reaction by using a luciferase kit (Thermo Fisher Scientific, Waltham, MA, USA). Chemiluminescent intensity was measured using a GloMax^®^-96 Microplate Luminometer (Promega Corporation, Madison, WI, USA) and normalized to protein concentrations.

### 2.9. Intracellular Accumulation of SBL_CD_-Luteolin

PC12 cells were treated with luteolin, SBL_CD_ or SBL_CD_-luteolin for 4 h and then washed with PBS and incubated with DPBA (diphenylboric acid 2-aminoethyl ester, 0.2% *w*/*v* in HBSS) for 15 min at room temperature in darkness for flavonoid localization [16]. Fluorescence intensity was measured using a FlexStation^®^3 multi-mode microplate reader (Molecular Devices, San Jose, CA, USA). For imaging, cells were co-stained with Mito-Tracker Red CMXRos (100 nM in HBSS) and DPBA, fixed with 4% paraformaldehyde, mounted with DAPI and imaged using a Leica SP8 confocal microscope (Leica Microsystems, Wetzlar, Germany at 63× magnification. Intracellular luteolin content was quantified by HPLC-MS/MS following 4 h treatment. Medium was collected and filtered through a 0.22 μm hydrophilic membrane. Cells were washed with PBS, harvested and sonicated in 70% acetonitrile for 30 min on ice. Cell debris was removed by centrifugation (3000 rpm, 3 min), and lysates were filtered through a 0.22 μm hydrophobic membrane for analysis. Luteolin content was normalized to protein concentration.

### 2.10. Statistical Analysis

In cell models, *n* = 4. Two-tailed unpaired *t*-test and one-way ANOVA with Bonferroni post hoc test were adopted for statistical analysis. The results with *p* < 0.05 were marked as statistically significant (*), and the results with *p* < 0.01 were marked as (**).

## 3. Results

### 3.1. Hydrothermal Synthesis and Optimization of SBL_CD_

**Figure 1:**
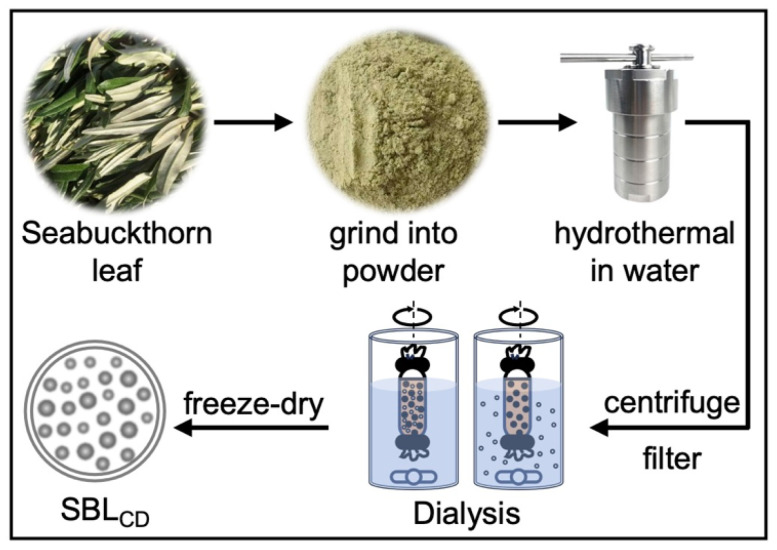
Preparation of carbon dots from Seabuckthorn leaves (SBL_CD_). Dried powder of Seabuckthorn leaf was dispersed in deionized water and hydrothermally treated at 180 °C for 5 h in a Teflon-lined autoclave. After natural cooling, the suspension was centrifuged and filtered to remove insoluble material, dialyzed against deionized water for 24 h (MWCO 500 Da) and freeze-dried to obtain SBL_CD_ powder for subsequent experiments.

Hydrothermal synthesis represents a green, cost-effective approach to produce carbon dots from various biomass sources, particularly fruit peels and plant residues [17,18]. The grounded powders of Seabuckthorn leaves were passed through a 100-mesh screen to achieve fine particles. The finalized hydrothermal conditions were determined based on preliminary trials evaluating temperature, time and water-to-biomass ratio, with the selection criteria including solution appearance and TEM characterization. The optimized condition was having 5 g fine powder dispersed in 100 mL deionized water in a 100 mL Teflon-lined autoclave, heated to 180 °C for 5 h in a blast air oven and then cooled naturally. The resultant was filtered through filter papers and centrifuged at 1500 rpm for 15 min to remove residues; the supernatant was then filtered through a 0.22 μm membrane. The obtained SBL_CD_ solution was dialyzed for 24 h against deionized water using a dialysis tube (500 Da MWCO) and freeze-dried for storage (Figure 1). The yield optimization resulted in an average production of 44 μg of freeze-dried SBL_CD_ per gram of dried leaf powder. When redissolved in DMSO at 1 mg/mL concentration, particle counting revealed 2.5 × 10^11^ particles per mL (analyzed by ZetaView^®^), indicating high particle density and uniform dispersion characteristics essential for biomedical applications. This corresponds to an average mass of approximately 4 × 10^−15^ g per particle and an effective nanoparticle molar concentration of ~4 × 10^−10^ M (assuming one particle as one entity).

### 3.2. Structural and Optical Characterization of SBL_CD_

**Figure 2:**
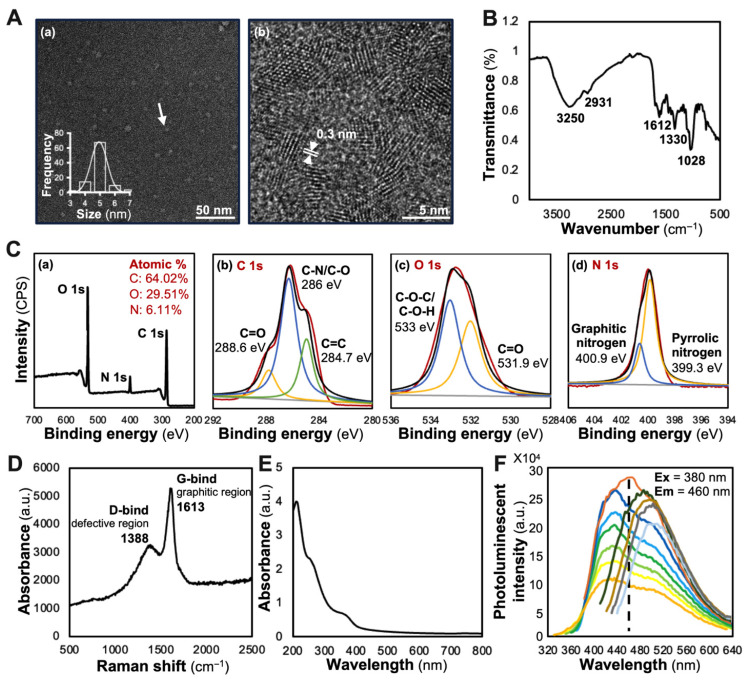
Structural and optical characterization of SBL_CD_. (**A**) Transmission electron microscopy (TEM) revealed uniformly dispersed nanoparticles with a mean diameter of ~5 nm (**a**, with white arrow indicating typical SBL_CD_ nanoparticles), and high-resolution TEM imaging shows lattice fringes with d-spacing ~0.3 nm (**b**). (**B**) FT-IR spectrum displayed O-H stretching (3250 cm^−1^), aliphatic C-H stretching (2931 cm^−1^), carbonyl/aromatic C=C stretching (1612 cm^−1^), C-N vibration (1330 cm^−1^) and C-O stretching (1028 cm^−1^). (**C**) XPS indicated elemental composition of C 64.02%, O 29.51% and N 6.11% (**a**), with high-resolution C1s peaks for *sp^2^* carbon (284.7 eV, green line), C-N/C-O (286 eV, red line) and carbonyl carbon (288.6 eV, orange line) (**b**); O1s peaks for ether/hydroxyl (533 eV, blue line) and carbonyl O (531.9 eV, orange line) (**c**); and N1s peaks for graphitic (400.9 eV, blue line) and pyrrolic (399.3 eV, orange line) nitrogen (**d**). (**D**) Raman spectrum showed the D-band at 1388 cm^−1^ (defects) and G-band at 1613 cm^−1^ (graphitic domains). (**E**) UV-visible absorption exhibited a main peak at 270 nm and a shoulder near 400 nm, characteristic of π-π* transitions in aromatic domains. (**F**) Photoluminescence spectra at 320–640 nm excitation displayed excitation-dependent emission, reaching a maximum emission intensity at 460 nm when excited at 380 nm.

Comprehensive characterization techniques, including TEM, FT-IR, Raman spectroscopy, UV-Vis absorption and photoluminescence analysis, provided detailed insights into the morphological and chemical structure of SBL_CD_. TEM analysis revealed that SBL_CD_ possessed a spherical morphology with a uniform nanoparticle exhibiting a narrow size distribution of 4–6 nm in diameter, as measured by ZetaView^®^ (Figure 2A(a)). High-resolution TEM imaging demonstrated the co-existence of amorphous and quasi-crystalline structures with lattice spacing of approximately 0.3 nm, consistent with well-characterized biomass-derived carbon dots [19] (Figure 2A(b)). FT-IR analysis identified key functional groups: O-H stretching vibration at 3250 cm^−1^, indicative of hydroxyl groups contributing to hydrophilicity and biocompatibility; aliphatic C-H stretching at 2931 cm^−1^; carbonyl/aromatic C=C stretching at 1612 cm^−1^; C-N vibration at 1330 cm^−1^; and C-O stretching vibrations at 1028 cm^−1^, suggesting the presence of alcohols, ethers, or phenolic compounds [20] (Figure 2B). XPS analysis confirmed an elemental composition of 64.02% carbon, 29.51% oxygen and 6.11% nitrogen, with a trace Si *2p* signal of ~0.35% from sample preparation; no metal impurities were detected above the instrumental detection limit. High-resolution XPS spectra revealed distinct carbon environments, C=C (284.7 eV), C-N/C-O (286.0 eV) and C=O/C=N (288.6 eV), with oxygen environments showing C=O (531.9 eV) and C-OH/C-O-C (533.0 eV) and nitrogen environments indicating graphitic nitrogen (400.9 eV) and pyrrolic nitrogen (399.3 eV) (Figure 2C).

Raman spectroscopy revealed distinctive structural features, with a D-band at 1388 cm^−1^ (defective regions) and G-band at 1613 cm^−1^ (graphitic regions), confirming the presence of both amorphous and quasi-crystalline forms [21,22]. The ID/IG ratio of 0.62 indicated a moderate degree of graphitization with a relatively ordered *sp^2^* carbon structure (Figure 2D). The UV-Vis spectral analysis of SBL_CD_ revealed two characteristic absorption features: a strong peak at 270 nm, corresponding to π-π* transitions of aromatic *sp^2^* domains in graphitic regions [23], and a weaker shoulder near 400 nm, attributed to n-π* transitions from surface oxygen-containing groups [24]. These dual absorption features indicated a hybrid structure of crystalline graphitic and amorphous carbon domains, typical of biomass-derived carbon dots [25] (Figure 2E). The excitation-dependent fluorescence emission spectrum demonstrated the strongest photoluminescence at 460 nm when excited at 380 nm, with intensity gradually decreasing as excitation wavelength increased, characteristic of carbon dots with excellent optical properties for bioimaging applications [26] (Figure 2F). These physical parameters provided characteristics of SBL_CD_.

### 3.3. Biocompatibility and Neuroprotective Properties of SBL_CD_

**Figure 3:**
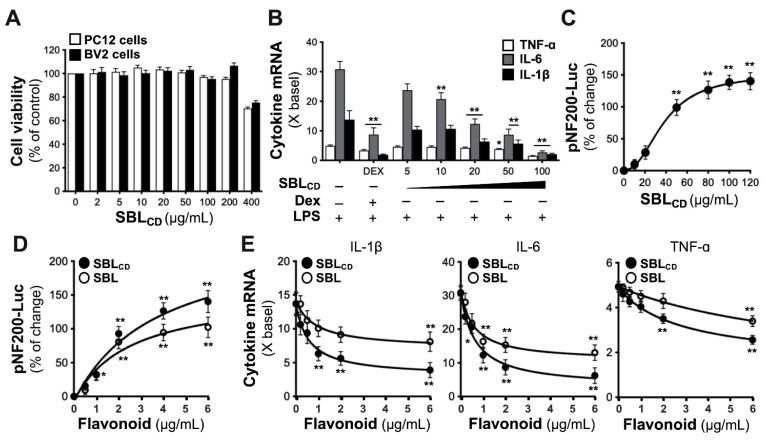
Comparative biological activities of SBL_CD_ against SBL. (**A**) MTT assay shows cell viability in cultured PC12 and BV2 cells after 0–400 μg/mL SBL_CD_ treatment. (**B**) BV2 cells pretreated with SBL_CD_ (2 h) exhibited dose-dependent suppression on LPS (20 ng/mL)-induced (16 h) IL-1β, IL-6 and TNF-α expressions. Cytokine mRNA levels were quantified by qRT-PCR and expressed as fold change (× basal) relative to the untreated control. Dexamethasone (10 μM) serves as a positive control. (**C**) In NF200-Luc DNA transfected PC12 cells, the activity of pNF200-Luc reporter was induced by 0–120 μg/mL SBL_CD_ in a dose-dependent manner. (**D**) At equivalent flavonoid concentrations (0–6 μg/mL), SBL_CD_ elicits greater induction of pNF200-Luc activity than that of Seabuckthorn leaf (SBL) extract in pNF200-Luc transfected PC12 cells. (**E**) Under the same flavonoid equivalents, SBL_CD_ shows stronger inhibition of LPS-induced cytokines in BV2 cells than SBL extract. Experimental procedure was performed as in (**B**). Values are presented as the percentage of control (%), the percentage of change (%) or the fold of change (× basal) to the control, in mean ± SEM, *n* = 4. (*) or (**) means the significance of changes between the control group and the SBL_CD_- or SBL- treated group. (*) *p* < 0.05, (**) *p* < 0.01.

SBL_CD_ demonstrated excellent biocompatibility in neuronal cell systems, with PC12 cells and BV2 cells maintaining viability >90% at concentrations up to 400 μg/mL, consistent with recent studies on biomass-derived carbon dots showing superior biocompatibility compared to synthetic nanomaterials [27] (Figure 3A).

Anti-inflammatory properties were assessed in cultured BV2 microglial cells treated with SBL_CD_ for 2 h prior to LPS stimulation (20 ng/mL). SBL_CD_ significantly suppressed LPS-induced expression of the pro-inflammatory cytokines IL-1β, IL-6 and TNF-α in dose-dependent manners, with dexamethasone serving as a positive control. Significance markers indicated the suppression of LPS-induced cytokine production by individual treatments versus the LPS treatment group (Figure 3B). To comprehensively evaluate the anti-inflammatory efficacy of SBL_CD_, inter-treatment differences were assessed. Quantitative analysis of maximal inhibition revealed that SBL_CD_ at 100 μM achieved 69.7% inhibition of TNF-α (compared to dexamethasone 31.7%, *p* < 0.001), 91.1% inhibition of IL-6 (compared to dexamethasone 71.7%, *p* < 0.001) and 84.9% inhibition of IL-1β (compared to dexamethasone 86.2%, *p* > 0.05), indicating that the effect of SBL_CD_ suppressing inflammation was more robust than that of dexamethasone. In addition, the capabilities of SBL_CD_ to induce neuronal differentiation were evaluated by using PC12 cells transfected with pNF200-Luc reporter construct, reflecting the transcriptional regulation of neurofilament [28]. The treatment of SBL_CD_ induced pNF200-Luc activity in a dose-dependent manner, achieving ~2.5-fold induction peak at 120 μg/mL concentration, demonstrating a robust capability to promoting neuronal differentiation (Figure 3C). These results demonstrate that SBL_CD_ possesses intrinsic bioactive properties, e.g., flavonoidic compounds within Seabuckthorn leaf, beyond simple drug carrier functions, consistent with recent findings on the therapeutic potential of carbon dots in neurological applications [29].

To specifically evaluate the advantage of nano-formulation beyond its flavonoid content, further experiments comparing the beneficial neuronal functions of extracts from Seabuckthorn leaf (SBL) and SBL_CD_ when containing the same amount of the targeted bioactive chemical, i.e., flavonoids, were performed. This comparison directly tests whether the nanoparticle itself enhances bioavailability and cellular accessibility. HPLC-MS/MS analysis revealed significant differences in flavonoid content between raw materials and processed SBL_CD_. The extract of Seabuckthorn leaves contained 35.69 mg total flavonoids per gram (dry weight), including 2.53 mg isorhamnetin, 1.35 mg quercetin and 1.23 mg kaempferol. Following hydrothermal synthesis, SBL_CD_ retained 7.85 mg total flavonoids per gram, comprising 0.71 mg isorhamnetin, 0.31 mg quercetin and 0.30 mg kaempferol. This represents approximately 22% retention of the original flavonoid content (Figure S1 and Table S3). The reduction in flavonoid content observed during hydrothermal carbon dot synthesis from Seabuckthorn leaf material resulted from multiple interconnected physicochemical processes occurring throughout the synthesis procedure. During hydrothermal processing at elevated temperatures approaching 180 °C, the flavonoids may undergo comprehensive chemical modifications, including dehydration reactions, carbonization processes and structural reorganization, contributing to the core formation of the carbon dot [30]. These transformations are characterized by distinctive Raman spectral signatures displaying D-band and G-band peaks at 1388 and 1613 cm^−1^, respectively (see Figure 2D), indicating the emergence of both amorphous and graphitic carbon structures. The thermal environment could induce degradation of thermolabile flavonoid molecules, with significant decomposition occurring above 150 °C, as documented in previous studies [31]. Furthermore, the surface functionalization mechanisms, evidenced by characteristic FT-IR absorption bands at 3250 cm^−1^ (-OH), 2931 cm^−1^ (C-H) and 1612 cm^−1^ (C=O/C=C) (see Figure 2B), should involve the conversion of intact flavonoid structures into diverse functional groups rather than preservation as complete molecular entities [24]. The XPS analysis revealed C-O, C=O and C-N bonding configurations, providing additional confirmation of these chemical modifications. Subsequently, the post-synthesis purification procedures, including centrifugation and filtration steps, could contribute to the elimination of water-soluble flavonoid components that failed to incorporate into the carbon dot matrix, as demonstrated by the homogeneous particle size distribution of 4–6 nm observed in TEM imaging [22]. Despite this quantitative reduction in total flavonoid content, the enhanced biological activity demonstrated by the remaining flavonoid components suggests improved bioavailability through the approach of nano-formulation.

At equivalent concentrations of total flavonoid, SBL_CD_ exhibited superior biological efficacy compared to conventional Seabuckthorn leaf extract (SBL) across multiple experimental paradigms. In PC12 neuronal cells transfected with pNF200-Luc reporter, the treatments with SBL or SBL_CD_ induced concentration-dependent increases in pNF200-Luc promoter activity, with SBL_CD_ demonstrating enhanced potency at the matched flavonoid concentrations (Figure 3C,D). Although both formulations exhibited comparable EC_50_ values (~1.30 μg/mL) for neurofilament promotion, SBL_CD_ achieved approximately 40% superior pNF200-Luc upregulation at 6 μg/mL flavonoid concentration. In LPS-challenged BV2 microglial cells, both SBL and SBL_CD_ exhibited concentration-dependent anti-inflammatory responses: SBL_CD_ enhanced the efficacy in suppressing pro-inflammatory cytokine expressions, e.g., IL-1β, IL-6 and TNF-α (Figure 3E). The superior potency of SBL_CD_ was demonstrated by the substantially reduced concentrations required to achieve comparable anti-inflammatory EC_50_ values. For IL-1β suppression, SBL_CD_ required only 0.19 μg/mL instead of 0.64 μg/mL for the conventional SBL extract. For IL-6 inhibition, SBL_CD_ required 0.18 μg/mL compared to 0.30 μg/mL for SBL; and for TNF-α suppression, SBL_CD_ required 1.02 μg/mL compared to 2.11 μg/mL for the conventional extract to achieve equivalent efficacy endpoints. These findings demonstrate that the nano-formulation could significantly enhance the biological activity of Seabuckthorn leaf flavonoids, requiring substantially lower concentrations in order to achieve equivalent or superior effects in both neuronal differentiation and anti-inflammatory responses.

### 3.4. Drug Loading and Enhanced Delivery Capabilities of SBL_CD_

**Figure 4:**
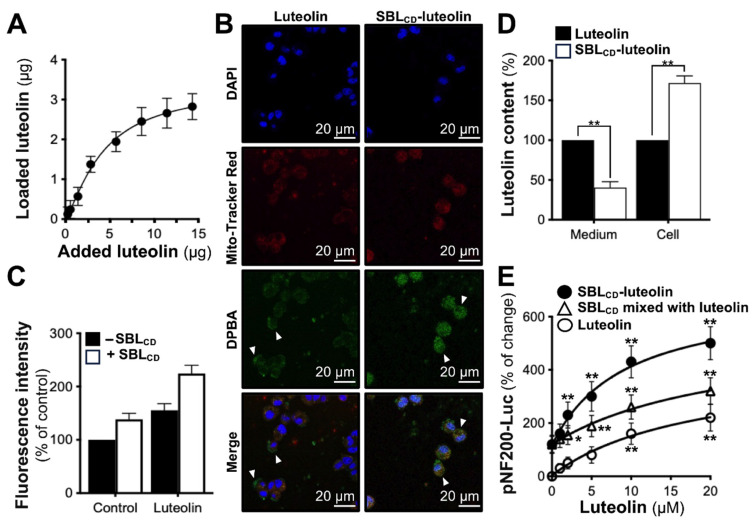
Luteolin loading capacity of SBL_CD_ and enhancement of cellular delivery. (**A**) Loading with of luteolin per 1 mg/mL SBL_CD_ reached a loading plateau of ~2.8 μg/mg. (**B**) Confocal images of PC12 cells after incubation with free luteolin or SBL_CD_-lutelin showed DPBA-labeled flavonoids (green), mitochondria (MitoTracker Red, red) and nuclei (DAPI, blue); arrowheads mark enhanced cytoplasmic accumulation in the SBL_CD_ group. (**C**) Fluorescence quantification confirmed higher intracellular luteolin with SBL_CD_ carriers than with free luteolin. (**D**) HPLC-MS/MS analysis showed greater luteolin uptake with SBL_CD_ delivery, normalized to cellular protein. (**E**) pNF200-Luc assay revealed a dose-dependent neurite promotion by free luteolin, SBL_CD_-luteolin and an SBL_CD_ + luteolin physical mixture; the complex outperforms both controls at all doses. Values are presented as the percentage of control (%) or the percentage of change (%), in mean ± SEM, *n* = 4. (*) or (**) means the significance of changes between the control group and the drug-treated group. (*) *p* < 0.05, (**) *p* < 0.01.

The amount of luteolin in Seabuckthorn leaf is very minimal. Thus, the drug delivery potential of SBL_CD_ in carrying luteolin was evaluated. Ultrasonication and ultrafiltration techniques were employed for encapsulation. Loading capacity studies demonstrated that the maximum loading of luteolin was approximately 2.8 μg per 1 mg SBL_CD_, determined through dose-dependent loading experiments, followed by HPLC-MS/MS analysis (Figure 4A). The loading at this value represented the plateau saturation, as further increases in luteolin concentration did not enhance the loading capacity. In addition, the intracellular localization studies using DPBA (diphenylboric acid 2-aminoethyl ester) fluorescent probe demonstrated the enhanced cellular uptake of SBL_CD_-luteolin as compared to free luteolin. As shown in Figure 4B, the confocal microscopy revealed that both luteolin and SBL_CD_-luteolin (green fluorescence) were co-localized with Mito-Tracker Red (mitochondrial marker), indicating successful cellular internalization. However, SBL_CD_-luteolin exhibited a significantly higher degree and intensity of co-localization, while free luteolin showed considerable green fluorescence outside the mitochondrial regions. Quantitative fluorescence analysis using a FlexStation^®^3 reader confirmed that SBL_CD_-luteolin treatment resulted in significantly increased fluorescence intensity as compared to equivalent concentrations of free luteolin (Figure 4C). HPLC-MS/MS quantification of intracellular luteolin content demonstrated notable enrichment in PC12 cells being treated with SBL_CD_-luteolin compared to free luteolin at equivalent concentrations (Figure 4D). The enhanced delivery efficiency translated to superior biological activity, with SBL_CD_-luteolin requiring only 1.50 μg/mL in achieving the EC_50_ efficacy as compared to 6.82 μg/mL for free luteolin in the pNF200-Luc expression assays. The physical mixture of SBL_CD_ and luteolin required an intermediate concentration of 3.14 μg/mL, confirming the specific advantages of the complex formulation over simple co-administration (Figure 4E).

## 4. Conclusions and Discussion

SBL_CD_ synthesized from Seabuckthorn leaf via a green hydrothermal process exhibited excellent biocompatibility (>90% cell viability) as well as superior neuroprotective and anti-inflammatory activities compared to the crude extract at equivalent flavonoid concentrations, indicating that nano-carbonization effectively enhanced the bioactivities of the plant-derived compounds. Efficient luteolin encapsulation (~2.8 μg/mg SBL_CD_) resulted in a ~4.5-fold enhancement of its potency (EC_50_: 1.50 μg/mL versus 6.82 μg/mL for free luteolin), demonstrating the advantage of nanoparticle-mediated intracellular delivery. These findings establish SBL_CD_ as a dual-function platform combining inherent therapeutic activity with enhanced drug carrier capacity.

SBL_CD_ exhibits several innovative aspects distinguishing it from existing carbon dot platforms. While most of the biomass-derived carbon dots utilize agricultural waste, e.g., fruit peels or vegetable residues [32], the synthesis of SBL_CD_ leverages the flavonoid-rich Seabuckthorn leaves that provide bioactive compounds alongside the carbon framework. Indeed, the leaf contains a 2–3-fold higher level of flavonoids as compared to the fruit. Unlike conventional carbon dots synthesized from citric acid or glucose precursors [33], SBL_CD_ contains intrinsic therapeutic properties, i.e., containing rich flavonoids, in creating a dual-function platform serving as both a nanocarrier and active pharmaceutical ingredients: this is a distinction to those pharmacologically inert carriers [34]. The traditional concept in natural products holds that the therapeutic functions can be retained even after the material is carbonized, as in the use of charcoal-processed remedies. Nanotechnology provides a unique bridge between empirical wisdom and contemporary science, offering rigorous validation for pharmaceutical applications through biomass-derived nanomaterials that enable sustainable, high-value utilization of agricultural by-products.

Structurally, SBL_CD_ shares several key features with other biomass-derived carbon dots reported in the literature, including a narrow particle size distribution from 2 to 10 nm, co-existence of amorphous and graphitic domains as well as abundant surface oxygen- and nitrogen-containing functional groups [35]. The observed D- and G-bands at 1388 and 1613 cm^−1^ with an ID/IG ratio of 0.62 indicate a moderately graphitized *sp^2^* carbon core with defect-rich regions, comparable to polyphenol-derived carbon dots from tea and other plant-based dots [36]. Likewise, FT-IR and XPS spectra of SBL_CD_ have revealed O-H, C=O/C=C, C-O and C-N surface groups and nitrogen species assigned to graphitic and pyrrolic N, which are typical of heteroatom-doped biomass carbon dots [37]. The strong *π-π* absorption at ~270 nm, with an *n-π* shoulder near 400 nm, together with emission peaking at 460 nm upon 380 nm excitation further recognize SBL_CD_ within the class of biomass-derived caron dots [38].

Compositionally, SBL_CD_ is distinguished by its nitrogen-enriched surface (6.11%) and unique flavonoid retention profile (~22% of original content), which significantly differentiates it from conventional carbohydrate-derived carbon dots or other fruit-derived carbon dots lacking quantifiable phytochemical signatures. The retained flavonoids likely interact with the carbon dot surface through *π-π* stacking and hydrogen bonding, creating a hybrid system where both the flavonoid cargo and the *sp^2^*-rich carbon scaffold synergistically enhance the bioavailability and cellular uptake. This mechanistic synergy is consistent with the observation here that SBL_CD_ outperformed the crude leaf extract, indicating that nanostructure-mediated delivery and surface interactions amplify the intrinsic bioactivity of retained flavonoids.

Moreover, the encapsulation ability of SBL_CD_ extended to other bioactive compounds. LC-MS analysis provided preliminary evidence suggesting SBL_CD_ possessed peptide loading capabilities (Figure S2). The chromatographic comparison revealed an additional peak in the SBL_CD_-peptide sample, indicating peptide may be effectively encapsulated through electrostatic interactions between SBL_CD_’s abundant surface functional groups and the charged peptide residues. Based on the retention of peptide spectral characteristics, it can be inferred that SBL_CD_ likely preserves the structural integrity of loaded biomolecules while acting as effective nanocarriers. The appearance of a delayed elution peak distinct from the free peptide, combined with concentration-dependent intensity increases for both of the peaks, suggested successful formation of peptide-nanoparticle complexes. These preliminary findings indicate promising peptide loading capabilities; however, comprehensive validation studies, including drug loading efficiency quantification, release kinetics and stability assessments, are necessary to fully characterize the therapeutic potential of this delivery system. The observed peptide-nanoparticle interactions warrant further investigation as a foundation to develop targeted drug delivery applications.

In a recent analysis, carbon dot-based protein delivery maintained up to 89% of the protein’s native biological activities [39]. The peptide loading capability opens possibilities for its applications in neurodegenerative disease, where peptide-based therapeutics often face delivery challenges [40]. Therefore, the ability to encapsulate and deliver both flavonoids and peptides highlights the multifunctional potential of SBL_CD_ as a versatile platform for the delivery of bioactive compounds. Although pristine carbon dots are smaller than the 4–6 nm gaps between endothelial tight junctions, Li et al. [41] demonstrated that passive diffusion alone is insufficient for blood-brain barrier (BBB) penetration and that receptor-mediated transcytosis (e.g., via transferrin conjugation) is required for effective CNS delivery. In parallel, the glucose-derived carbon dots demonstrated a promising BBB penetration through glucose transporter-dependent mechanisms, effectively accumulating in the CNS tissues, which was proposed in delivering drugs to the brain [42]. The small size (~5 nm) of SBL_CD_, combined with multifunctional surface properties, could facilitate BBB penetration through receptor-mediated mechanisms, potentially enabling drug delivery to brain tissues where conventional therapeutics often fail.

At the mechanistic level, the observed neuroprotective and anti-inflammatory effects of SBL_CD_ were consistent with the bioactivity of flavonoids, as determined in Seabuckthorn leaves. Previous work demonstrated that Seabuckthorn flavonoids promoted neuronal differentiation through PI3K/Akt and ERK signaling, as confirmed by pharmacological inhibition with LY294002 and U0126 [12,13]. These signaling cascades could be applied similarly to those of SBL_CD_ bioactivities. The enhanced intracellular luteolin accumulation upon SBL_CD_ loading is consistent with nanoparticle-mediated endocytic uptake rather than passive diffusion, as carbon dots are typically internalized via clathrin- and caveolae-mediated endocytosis [43]. Future studies employing pathway-specific inhibitors (LY294002 and U0126) could be performed to validate these mechanisms.

This study acknowledges several limitations. The low gravimetric yield (0.0044%) reflects stringent purification and is typical for biomass-derived carbon dots (<1% recovery) [44]. From a mass-balance perspective, this low gravimetric yield reflects the fact that only a minor fraction of the leaf biomass is converted into highly dispersed, photoluminescent nanodots, whereas the majority of precursor carbon remains as insoluble hydrochars or dialyzable small molecules that are being removed during purification. Although hydrothermal processing reduces total flavonoid content by 78%, the retained flavonoids exhibit a 3~4-fold enhanced potency per unit mass, resulting in comparable or superior bioactivity compared to the original extract. Spectroscopy revealed carbogenic structures; however, the distribution among intact flavonoids, carbonized intermediates and carbon dot properties remains unclear. Additionally, the synthesis reproducibility showed significant limitations due to batch-to-batch variations from plant material heterogenicity [45]. The heterogenicity of herbal materials generates the problems of standardization and quality control, particularly, the stringent requirements for pharmaceutical consistency of batch variation [46]. Moreover, regulatory requirements present additional challenges, as the current nanomedicine regulations lack specific guidelines for biomass-derived carbon dots, creating uncertainty for the approval process [47].

The comprehensive characterization of drug loading and release behavior is essential for translating carbon dot-based carriers into clinically viable drug delivery platforms. Key parameters including loading efficiency, encapsulation efficiency (%), in vitro release profiles under physiological conditions (PBS, serum, variable pH) and physicochemical stability over 24–72 h should be systematically evaluated in the future study. These parameters enable the assessment of drug-carrier interactions, prediction of in vivo pharmacokinetics and optimization of therapeutic efficacy [48,49].

## Data Availability

The original contributions presented in the study are included in the article, further inquiries can be directed to the corresponding author.

## Supplementary Materials

The following supporting information can be downloaded at: https://www.mdpi.com/article/10.3390/jfb16120465/s1.
