# Supplementary material for: The Carbon Dots from Seabuckthorn (Hippophae rhamnoides L.) Leaves: Recycle the Herbal Waste Products for a Nano-Formulation in Delivering Bioactive Compounds
Source: J Funct Biomater. 2025 Dec 17;16(12):465. doi: 10.3390/jfb16120465 (PMC12734045; doi:10.3390/jfb16120465)
Supplement: Supplementary file 1 [file jfb-16-00465-s001.zip › jfb-3984756-supplementary.pdf]

Journal of
Functional
Biomaterials

MDPI

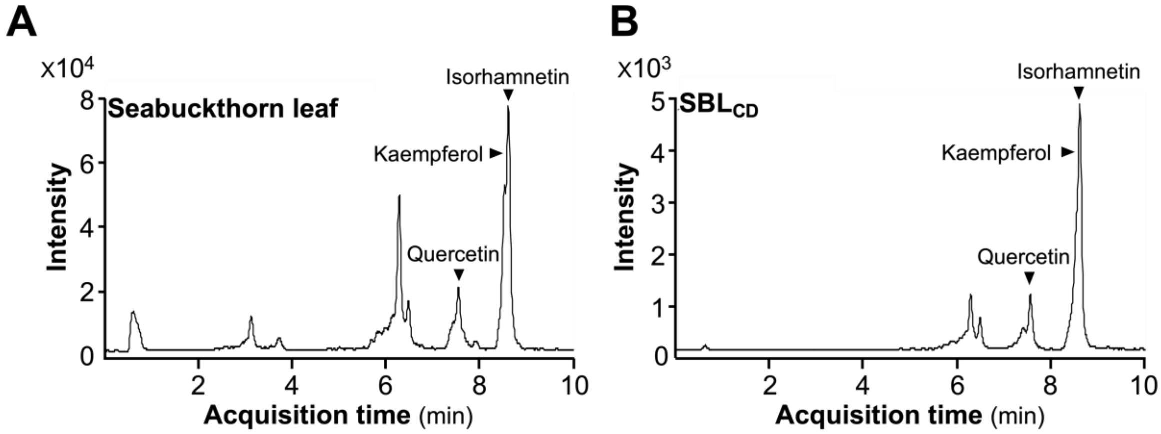

Figure S1. Chemical characterization of Seabuckthorn leaf and SBL$_{CD}$. The flavonoid composition in Seabuckthorn leaf extract **(A)** and SBL$_{CD}$ **(B)** was determined using HPLC-MS/MS with isorhamnetin, quercetin and kaempferol as reference standards. Chromatographic separation was achieved using an Agilent ZORBAX Eclipse Plus C$_{18}$ Column (2.1 × 50 mm, 1.8 μm) with a gradient elution: solvent A (0.1% formic acid in H_2O) and solvent B (0.1% formic acid in acetonitrile). The gradient profile was as follows: 0-20 min, 2% B; 20-20.01 min, 100% B; 20.01-25 min, 2% B. Injected sample volume was 5 μL at a flow rate of 0.3 mL/min. Quantitative analysis was performed using Multiple Reaction Monitoring (MRM) mode on an Agilent Triple Quadrupole Tandem Mass Spectrometer (QQQ-MS/MS, 6410A).

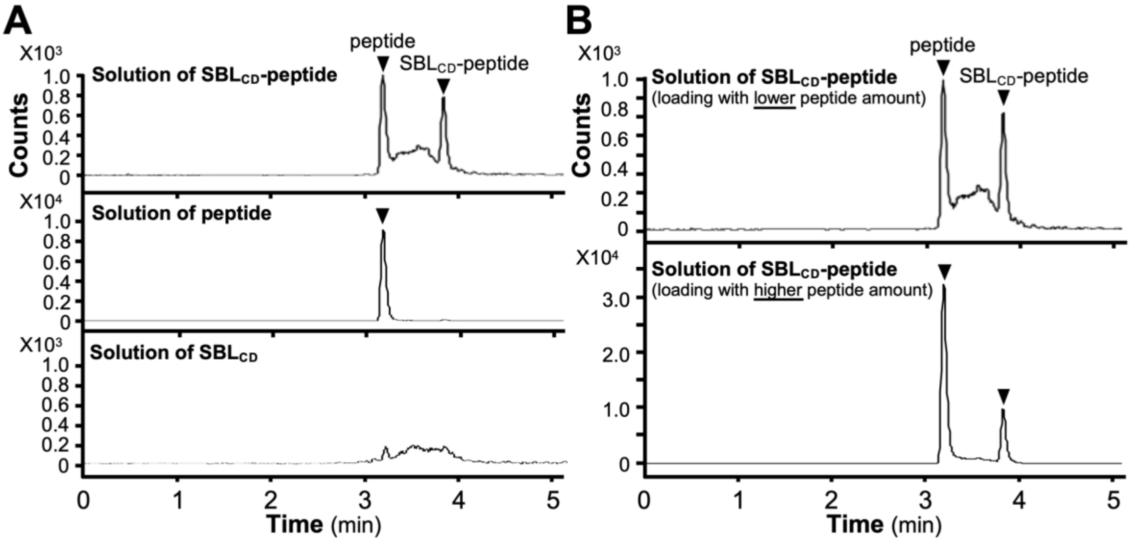

Figure S2. LC-MS confirmation of peptide loading on SBL$_{CD}$. A heptapeptide (molecular weight: 685.3547 Da having a sequence of LAG AAH F)) derived from pigment epithelium-derived factor was loaded onto SBL$_{CD}$ in a 1:1 ratio (w/w) through continuous rotation for 24 h at 4°C, followed by dialysis purification (1 kDa MWCO) for 24 hours. **(A)** LC chromatograms obtained using Agilent ZORBAX Eclipse Plus C$_{18}$ Column (2.1 x 50 mm, 1.8 μm) with mobile phases of 0.1% formic acid in H_2O (solvent A) and 0.1% formic acid in acetonitrile (solvent B) at 0.3 mL/min flow rate. Peptide alone (middle) showed a single peak at ~3 min, whereas SBL$_{CD}$-peptide complex (top) presented this peak plus a later eluting peak at ~4 min corresponding to the complex formation. SBL$_{CD}$ solution alone (bottom) showed only minor background peaks at the detection wavelength optimized for

peptide absorption, confirming that the prominent peaks observed are not derived from SBL_{CD} interference. **(B)** Different peptide concentrations (lower and higher amounts) were loaded into identical 1 mg/mL SBL_{CD} solution. As peptide loading increased, both the early eluting peptide peak (~3 min) and the delayed peak (~4 min) showed proportional intensity increases, providing evidence that the later-eluting peak corresponds to peptide-nanoparticle complexes rather than unrelated impurities or artifacts.

Supplementary Table S1. Optimized HPLC-MS/MS scan segments

Target	Precursor ion	Product ion	Fragmentor voltage (V)	Collision energy (V)
Isorhamnetin	315.1	151/300	150	13/21
Quercetin	301.0	151/107	100	13/21
Kaempferol	285.0	117/93	150	29/41
Luteolin	286.1	151/133	150	21/33

Supplementary Table S2. Primers used in qRT-PCR analysis

Primer	Sequence (5'-3')	
	Forward	Reverse
GAPDH	CATCACTGCCACCCAGAAGACTG	ATGCCAGTGAGCTTCCCGTTCAG
IL-1β	GCAACTGTTCCTGAACTCAACT	ATCTTTTGGGGTCCGTCAACT
IL-6	TAGTCCTTCCTACCCCAATTTCC	TTGGTCCTTAGCCACTCCTTC
TNF-α	CCTGTAGCCCACGTCGTAG	GGGAGTAGACAAGGTACAACCC

Supplementary Table S3. Chemical characterization of SBL and SBLCD.

Origin	Quercetin (mg/g)[a]	Kaempferol (mg/g)[a]	Isorhamnetin (mg/g)[a]	Total flavonoids (mg/g)[b]
**SBL**	1.352 ± 0.024	1.233 ± 0.023	2.525 ± 0.040	35.688 ± 0.889
**SBL_{CD}**	0.305 ± 0.006	0.301 ± 0.008	0.707 ± 0.025	7.851 ± 0.186

Single flavonoid contents (i.e., quercetin, kaempferol and isorhamnetin) in Seabuckthorn leaves (SBL) and SBL_{CD} were quantified using HPLC-MS/MS with an Agilent ZORBAX Eclipse Plus C_{18} Column (2.1 × 50 mm, 1.8 μm). Total flavonoid content was determined by UV-Vis spectroscopy.

[a] The values are measured from HPLC-MS/MS analysis in mean ± SEM, *n* = 4.

[b] The values are measured from UV-Vis analysis in mean ± SEM, *n* = 4.
